# Chromosomal localization of genes conferring desirable agronomic traits from *Agropyron cristatum* chromosome 1P

**DOI:** 10.1371/journal.pone.0175265

**Published:** 2017-04-10

**Authors:** Cuili Pan, Qingfeng Li, Yuqing Lu, Jinpeng Zhang, Xinming Yang, Xiuquan Li, Lihui Li, Weihua Liu

**Affiliations:** 1 National Key Facility for Crop Gene Resources and Genetic Improvement, Institute of Crop Science, Chinese Academy of Agricultural Sciences, Beijing, China; 2 College of Agronomy, Northwest A&F University, Yangling, Shaanxi, China; Institute of Genetics and Developmental Biology Chinese Academy of Sciences, CHINA

## Abstract

*Agropyron cristatum* (L.) Gaertn. (2n = 4x = 28, PPPP), a wild relative of common wheat, possesses many potentially valuable genes for wheat breeding. To transfer these genes into wheat, a series of wheat-*A*. *cristatum* derivatives have been obtained in our laboratory. In this study, a wheat-*A*. *cristatum* derivative II-3-1 was obtained, which was proven to contain a 1P (1A) disomic substitution and 2P disomic addition line with 40 wheat chromosomes and two pairs of *A*. *cristatum* chromosomes by genomic in situ hybridization (GISH) and molecular markers analysis. By further backcrossing with the wheat parent Fukuhokomugi (Fukuho) and self-fertilization, three different lines were separated from II-3-1, including wheat-*A*. *cristatum* 1P disomic addition line II-3-1a, 2P disomic addition line II-3-1b and 1P (1A) disomic substitution line II-3-1c. Because 2P addition line had been reported before, we aimed to investigate 1P disomic addition line II-3-1a and wheat-*A*. *cristatum* 1P (1A) disomic substitution line II-3-1c. Analysis of different genetic populations demonstrated that 1P chromosome harbored multiple agronomic traits, such as elevated spike length, increased tillering ability, reduced plant height and spikelet density. Besides, bristles on the glume ridges as an important morphological marker was located on 1P chromosome. Therefore, the novel 1P addition and substitution lines will be used as important genetic materials to widen the genetic resources of wheat.

## Introduction

Wild relatives of wheat belonging to tertiary gene pools are valuable sources of new genetic variation for wheat improvement [1]. *Agropyron* genus, which mainly includes *A*. *cristatum* (L.) Gaertn, *A*. *deserlorum* (Fiseh.) Schult, *A*. *fragile* (Roth.) Candargy, *A*. *michnoi* Roshev. and *A*. *mongolicum* Keng, is an important genus related to wheat and has three ploidy levels in nature, i.e. diploid (2n = 2x = 14, PP), tetraploid (2n = 4x = 28, PPPP) and hexaploid (2n = 6x = 42, PPPPPP) [2]. *A*. *cristatum* is the most common species of *Agropyron* genus distributed on grasslands and sands of Eurasian low-temperature regions [2]. It contains many excellent characteristics. For example, resistance to wheat take-all fungus, stripe rust, and powdery mildew [3–5]; tolerance to drought and salinity [6]; and superior numbers of florets and tillers [7, 8].

To utilize the desirable genes of *A*. *cristatum* for wheat improvement, plant breeders started to study hybridization between wheat and *Agropyron* genus in the 1940s, and successes have not been made until the 1980s [3, 9–11]. Li et al. [12, 13] not only obtained the hybrids between *A*. *cristatum* L. Gaertn. and common wheat, but also obtained a series of stable wheat-*A*. *cristatum* addition lines and translocation lines, such as 2P disomic addition line and translocation line [5, 14], 6P disomic addition line, substitution line and translocation line [4, 7, 8, 15–17], and 7P disomic addition line and translocation line [18]. However, 1P, 3P, 4P and 5P addition lines have not been reported. This study aims to produce wheat-*A*. *cristatum* 1P addition and substitution lines, and locate excellent agronomic characters on *A*. *cristatum* 1P chromosome.

Wide hybridization is always being used to transfer desirable traits from wild relatives to common wheat, creating germplasms with useful alien genes [19]. The disomic addition and substitution lines function as bridge tools to research the chromosomal genetic effect under wheat background, and produce useful translocation lines for wheat improvement. Therefore, detecting whether alien chromosomes have been transferred into wheat background is extremely important. Genomic in situ hybridization (GISH) and fluorescence in situ hybridization (FISH) are efficient and accurate methods to detect alien chromatin and allocate alien chromosomes on different homoeologous groups in common wheat [20], but it is difficult to genotype large genetic population or detect chromosomal fragment smaller than 10kb. In this case, molecular markers could be used to instead of cytological methods. For example, Sharp et al. [21] used RFLP to analyze the homoeology of alien chromosomes added to wheat; Li et al. [22] developed 37 specific-locus amplified fragment sequencing (SLAF) markers specific to chromosome 1St#2 used to trace specific *Th*. *intermedium* chromosomes under wheat background. A large number of markers specific to *A*. *cristatum* chromosomes had been developed in our laboratory, such as RAPD, EST-STS and repeat sequence probes [23–25], which lay good foundation for the identification of *A*. *cristatum* chromosomes.

The aims of the present study were: (1) to analyze the chromosomal constitution of wheat-*A*. *cristatum* derivative II-3-1; (2) to develop wheat-*A*. *cristatum* 1P disomic addition line and 1P (1A) substitution line; (3) to evaluate the desirable genes on *A*. *cristatum* 1P chromosome.

## Materials and methods

### Plant materials

Common wheat cv. Fukuho and *Agropyron cristatum* cv. Z559 were used as recipient parent and control, respectively. II-3-1 was identified from the F_3_ progenies of Z559 and Fukuho. II-3-1a and II-3-1b were isolated from the BC_2_F_2_ derived from the hybridization between II-3-1 and Fukuho. II-3-1 was backcrossed with common wheat Fukuho firstly, followed by the self-pollination for three times. II-3-1c was selected from F_4_ generation. The genetic populations BC_1_F_2_ and BC_2_F_2_ were from then backcrossing and self-pollination of II-3-1a and Fukuho. All the materials were developed and provided by the Center of Crop Germplasm Resources Research in the Institute of Crop Science, Chinese Academy of Agricultural Sciences (CAAS), Beijing, China.

### Cytological analyses of mitosis and meiosis

Chromosome preparations of root tips were made according to the previously described procedure [26]. For the pollen mother cells meiosis studies, the procedures were followed by Jauhar and Peterson [27].

### Genome In Situ Hybridization (GISH)

The alien *A*. *cristatum* chromosomes were detected by GISH using *A*. *cristatum* cv. Z559 genome DNA as probe and common wheat cv. Fukuho genome DNA as blocker. The total genomic DNA of common wheat Fukuho and *A*. *cristatum* Z559 were isolated using a modified CTAB method [28]. The GISH procedure was described by Liu et al. [29].

### Fluorescence In Situ Hybridization (FISH)

Probe pAs1 from *Aegilops tauschii* Coss. combined with probe pHvG39 from barley could distinguish A, B and D genome of common wheat [30]. *A*. *cristatum* repeats sequence pAcTRT1 and pAcpCR2 probes presenting different signals on different *A*. *cristatum* chromosomes could detect which *A*. *cristatum* chromosomes was included [24]. FISH procedure was described by Luan et al. [15]. All cytological images were observed under an OLYMPUS AX80 (Olympus Corporation, Tokyo, Japan) fluorescence microscope and captured with a CCD camera (Diagnostic Instruments, Inc., Sterling Heights, MI, USA).

### Molecular markers analysis

In order to identify alien chromosomes and determine homoeologous relationships between *A*. *cristatum* and wheat chromosomes in II-3-1, II-3-1a, II-3-1b, and II-3-1c, a total of 92 *A*. *cristatum* EST-STS markers from *A*. *cristatum* transcriptome sequences that corresponding to wheat homoeologous group 1 and 2 were used [25]. To detect presence/absence of wheat chromosomes in II-3-1 and II-3-1c, 33 wheat SSR markers located on wheat chromosome 1A, 1B and 1D were screened from the GrainGenes 2.0 website (https://wheat.pw.usda.gov/GG3/). All the information of primers was listed in S1 and S2 Tables. PCR amplification procedure and electrophoresis were described by Luan et al. [15].

### Evaluation of agronomic traits

The addition line II-3-1a, substitution line II-3-1c, and their parents II-3-1 and Fukuho were planted at the experimental farm of Xinxiang of Henan province in China in two sowing seasons (2014–2015 and 2015–2016). All the materials were planted in the randomized block arrangement with three repeats with spacing 30 cm apart and rows 2.0 m long with 20 grains per row. Plant height, spike length, spikelets per spike, kernels per middle spikelet, kernels per main spike, fertile tiller numbers, thousand grain weight and bristles on the glume ridges were evaluated. Similarly, two populations developed from continuous backcrosses and selfcrosses of II-3-1a and the recurrent parent Fukuho. These populations were planted in the 2014–2015 and 2015–2016 growing season to evaluate the key agronomic traits. Each individual was identified with the P genome-specific STS markers, and each population was then divided into two groups according to the absence of *A*. *cristatum* 1P specific markers. All the traits were measured on each plant from the segregating populations and on 20 plants randomly selected from the parents II-3-1, II-3-1a, II-3-1c and Fukuho. The data was analyzed by the Statistical Analysis System version 9.2 (SAS Institute Inc., Cary, NC, USA) adopting Duncan’s multiple range tests analysis of variance at the P = 0.05 significance levels.

## Results

### Chromosome composition analysis of II-3-1

**Fig 1:**
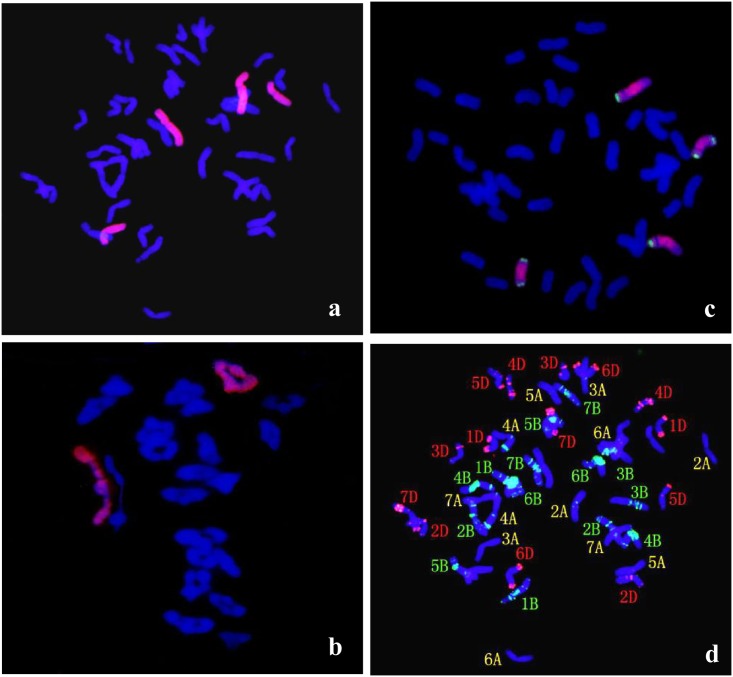
GISH-FISH patterns of the root cells and pollen mother cells at meiotic metaphase I of II-3-1. (a) GISH analysis of II-3-1 showing red hybridization signals evenly distributed on P chromosomes with *A*. *cristatum* genomic DNA as the probe and Fukuho DNA as the blocker. Wheat chromosomes were counterstained with DAPI (blue). (b) Pollen mother cells of II-3-1 at meiotic metaphase I. (c) FISH detection of chromosome 1P and 2P using pAcTRT1 and pAcpCR2 as probes. (d) FISH detection using pAs1 (red) and pHvG38 (green) repetitive DNA as probes showing that 1A chromosomes are missing in II-3-1.

Chromosome counting and GISH analysis showed that there were 44 chromosomes in the somatic cells of II-3-1, including 40 wheat chromosomes (shown in blue) and 4 *A*. *cristatum* chromosomes (shown in red) (Fig 1a). Chromosomal configurations at the metaphase I of pollen mother cells were counted in II-3-1, showing 2n = 22 II, with averages of 0.36 univalents, 2.18 rod bivalents, 19.55 ring bivalents, and 0.06 trivalents (Table 1). The presence of twenty wheat bivalents (shown in blue) and two *A*. *cristatum* bivalents (shown in red) suggested that the behaviors of chromosome pairing were regular (Fig 1b). All the results indicated that II-3-1containing two pairs of *A*. *cristatum* chromosomes was cytologically stable.

**Table 1:** Chromosome numbers in somatic cells and configuration at meiotic metaphase I in pollen mother cells of II-3-1, II-3-1a, II-3-1c and Fukuho.

Materials	2n	No. of cells	Chromosome configuration				
			Univalent	Bivalent			trivalent
				Rod	Ring	Total	
**II-3-1**	44	120	0.36	2.18	19.55	21.73	0.06
			(0–3)	(1–4)	(18–22)	(21–22)	(0–1)
**II-3-1a**	44	120	0.60	2.29	19.41	21.70	
			(0–2)	(0–3)	(19–22)	(21–22)	
**II-3-1c**	42	120	0.16	1.23	19.60	20.84	0.05
			(0–2)	(1–3)	(19–21)	(20–21)	(0–1)
**Fukuho**	42	100	0.06	1.95	19.32	20.97	
			(0–2)	(1–4)	(17–21)	(20–21)	

**Fig 2:**
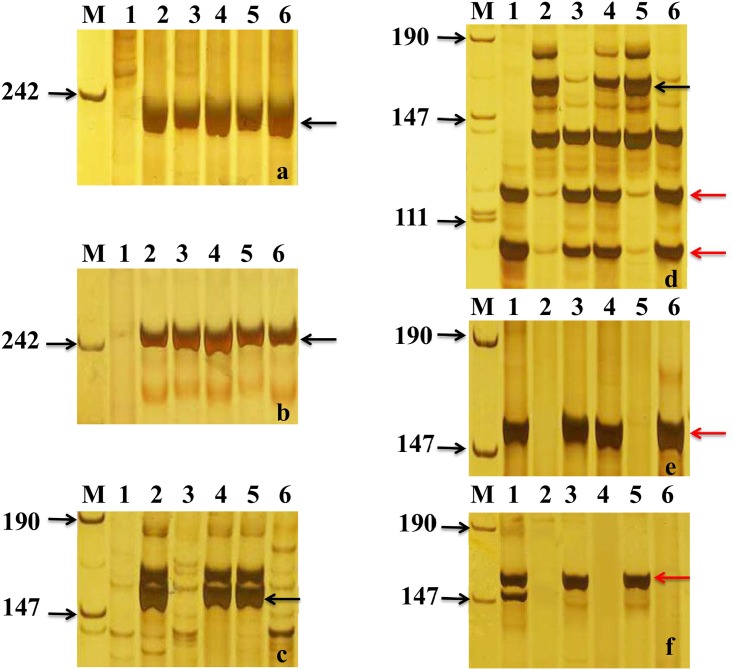
Amplification patterns of *A*. *cristatum* EST-STS markers, wheat SSR markers. (a, b, c) SSR markers gwm458, cfd92, and wmc278 are specific to 1B, 1D and 1A respectively; (d) SSR marker barc287 show polymorphism between 1A and 1P; (e, f) marker Agc9509 and Agc26072 are specific to 1P and 2P respectively. M, marker; lane1, *A*. *cristatum* cv. Z559; lane 2, *Triticum aestivum* cv. Fukuho; lane 3, II-3-1; lane 4, II-3-1a; lane 5, II-3-1b; lane 6, II-3-1c. The black arrows indicate wheat specific bands and the red arrows indicate P chromosomes specific bands.

FISH using pAcTRT1 and pAcpCR2 as probes demonstrated that the two pairs of chromosomes added to II-3-1 were 1P and 2P, respectively (Fig 1c). In order to detect which wheat chromosomes were absent in II-3-1, GISH-FISH and wheat SSR markers were used. GISH-FISH results demonstrated that a pair of *A*. *cristatum* chromosomes were replaced with a pair of wheat 1A chromosomes (Fig 1d). Thirty-three wheat SSR markers on wheat 1A, 1B and 1D were chose to further confirm detect which wheat chromosomes were absent in II-3-1. The results showed that 6 markers on chromosome 1B and 9 markers on chromosome 1D displayed specific bands in II-3-1 (Fig 2a and 2b), while 18 markers on chromosome 1A displayed no specific bands (Fig 2c). This result was consistent with the result of GISH-FISH identification, both of which indicated that the 1A chromosomes were substituted in II-3-1. In addition, three 1A-specific markers (barc287, cfe267, and cfe77) polymorphism between chromosomes 1P and 1A were screened (Fig 2d), suggesting that the added alien chromosomes and the absent wheat chromosomes belonged to the same homoelogous group. What’s more, fifty-six *A*. *cristatum* EST-STS markers aligned to wheat homoeologous group 1 and thirty-six aligned to wheat homoeologous group 2 can both amplified specific products in II-3-1 (Fig 2e and 2f). In conclusion, wheat-*A*. *cristatum* derivative II-3-1 contained a 1P (1A) disomic substitution and 2P disomic addition line.

### Production and molecular cytogengtic identification of 1P chromosomal addition line

**Fig 3:**
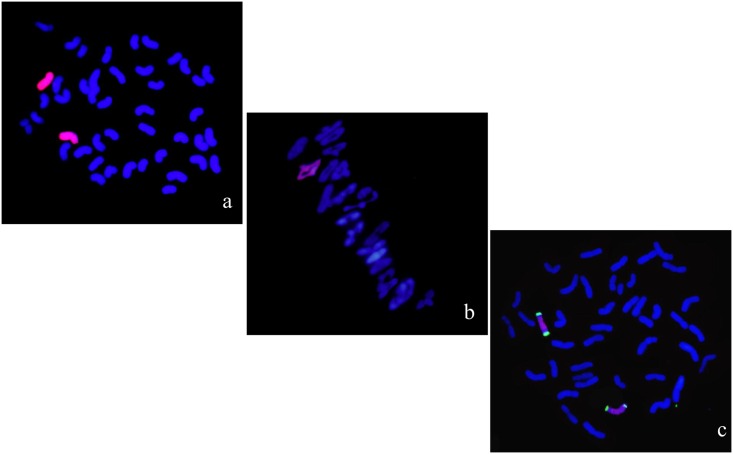
GISH-FISH patterns of the root cells and pollen mother cells at meiotic metaphase I of II-3-1a. (a) GISH analysis of II-3-1a showing red hybridization signals evenly distributed on P chromosomes with *A*. *cristatum* genomic DNA as the probe and Fukuho DNA as the blocker. Wheat chromosomes were counterstained with DAPI (blue). (b) Pollen mother cells of II-3-1a at meiotic metaphase I. (c) FISH detection of chromosome 1P and 2P using pAcTRT1 and pAcpCR2 as probes.

II-3-1a was obtained from the BC_2_F_2_ progenies of II-3-1 and Fukuho (Table 1). They formed 22 bivalents with an average pairing frequency of 2n = 44 = 0.6 I + 2.29 rod II + 19.41 ring II during meiotic metaphase I. GISH results of somatic cells showed that they contained 44 chromosomes including 42 wheat chromosomes and 2 of *A*. *cristatum* (Fig 3a and 3b). FISH identification with pAcTRT1 and pAcpCR2 as probes showed that the pair of chromosomes added were *A*. *cristatum* 1P (Fig 3c). Fifty-six *A*. *cristatum* EST-STS markers of group 1 also gave specific products to II-3-1a (Fig 2e). Those markers specific to 1P could not only be used to trace and identify chromosome 1P but also locate desirable genes.

### Production and molecular cytogengtic identification of 1P chromosomal substitution line

**Fig 4:**
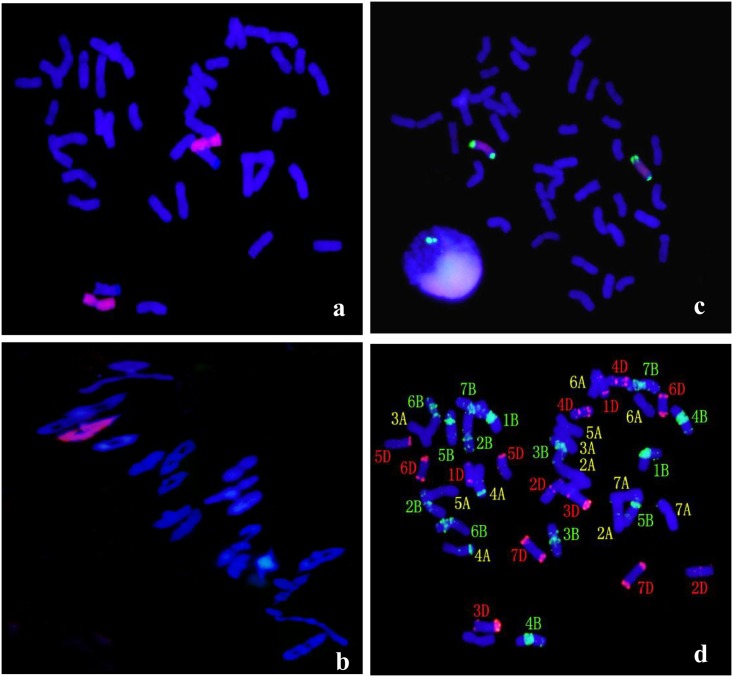
GISH-FISH patterns of the root cells and pollen mother cells at meiotic metaphase I of II-3-1c. (a) GISH analysis of II-3-1c showing red hybridization signals evenly distributed on P chromosomes with *A*. *cristatum* genomic DNA as the probe and Fukuho DNA as the blocker. Wheat chromosomes were counterstained with DAPI (blue). (b) Pollen mother cells of II-3-1c at meiotic metaphase I. (c) FISH detection of chromosome 1P and 2P use pAcTRT1 and pAcpCR2 as probes. (d) FISH using pAs1 (red) and pHvG38 (green) repetitive DNA as probes showing that 1A chromosomes are missing in II-3-1c.

II-3-1c was identified from the F_4_ progenies of II-3-1 (Fig 4a). Chromosome configuration at meiotic MI of PMCs showed 21 bivalents with an average pairing frequency of 2n = 42 = 0.16 I + 1.23 rod II + 19.60 II ring + 0.05 trivalents during meiotic metaphase I (Fig 4b) (Table 1). FISH identification with pAcTRT1 and pAcpCR2 probes demonstrated that the two *A*. *cristatum* chromosomes in II-3-1c were a pair of *A*. *cristatum* 1P chromosomes (Fig 4c). All the fifty-six EST-STS markers specific to chromosome 1P could be amplified characteristic products in II-3-1c (Fig 2e). GISH-FISH identification indicated that the substituted wheat chromosomes were a pair of chromosomes 1A (Fig 4d). The molecular cytogengtic identification suggested that II-3-1c was a stable wheat-*A*. *cristatum* 1P (1A) substitution line.

### The analysis of desirable genes for wheat improvement on chromosome 1P

**Fig 5:**
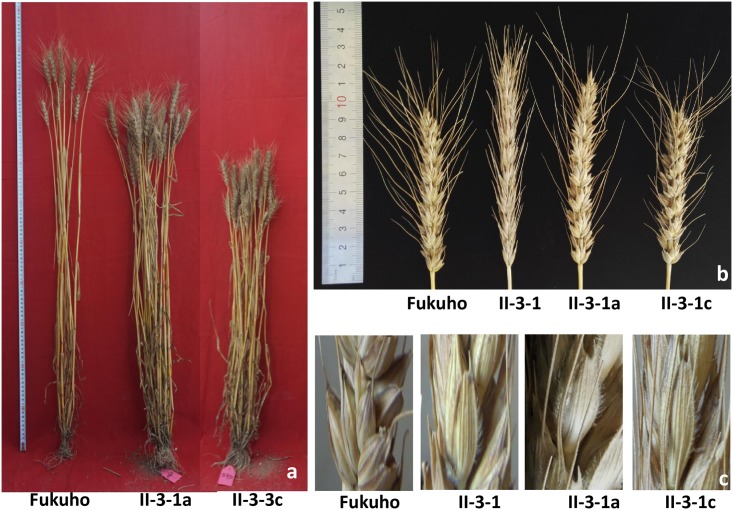
Morphological traits of II-3-1, the addition line II-3-1a, substitution line II-3-1c and Fukuho. a, Plant hieght; b, Spike length; c, Glume ridges bristles.

The statistic analysis suggested that 1P addition line II-3-1a differed from Fukuho in plant height (Fig 5a), spike length (Fig 5b), spikelet density, effective tiller numbers and the bristles on the glume ridges (Fig 5c) in the sowing years 2014–2015 and 2015–2016 (Table 2). In order to define the source of these traits, 1P addition line II-3-1a was crossed with common wheat Fukuho to construct BC_1_F_2_ and BC_2_F_2_ populations. In BC_1_F_2_ and BC_2_F_2_ populations, the average height of plants with chromosome 1P were 89.55 cm and 75.85 cm, respectively, significantly lower than those without 1P which were 94.41 cm and 80.00 cm, respectively. The average spikelet density of plants carrying chromosome 1P were 15.58 and 16.58, respectively, significantly lower than those lack of chromosome 1P which were 17.20 and 18.37, respectively. The average spike length of plants with chromosome 1P were 11.12 cm and 10.75 cm, respectively, significantly longer than those without chromosome 1P which were 9.85 cm and 9.66 cm, respectively. The average tiller numbers of plants carrying chromosome 1P were 23.18 and 18.00, respectively, more than those lack of chromosomes 1P which were 18.28 and 13.73, respectively. The bristles were present on the glume ridges in the plants with chromosome 1P and absent in the plants without 1P chromosomes, confirming that the gene controlling bristles on the glume ridges were located on chromosome 1P. The results above confirmed that the variation of plant height, spike length, spikelet density, tiller numbers and bristles on the glume ridges were affected by chromosome 1P. Therefore, wheat-*A*. *cristatum* 1P addition line II-3-1a could be a potential breeding material to reduce plant height and spikelet density, and to improve spike length and tillering ability. Additionally, the presence of bristles on the glume ridges was a typical characteristic of chromosome 1P which could be used to identify and trace the plants carrying 1P chromosome in segregating progenies. The wheat-*Agropyron cristatum* 1P addition line II-3-1a and 1P (1A) substitution line II-3-1c could provide novel genetic resources for producing translocation line for wheat improvement.

**Table 2:** Evaluation of agronomic traits of II-3-1a, II-3-1c, Fukuho and genetic populations derived from II-3-1a and Fukuho.

Year	Materials	PH(cm)	SL(cm)	SNPS	SD	KNMS	GNPS	FTN	TGW(g)	WGB
**2015**	**Fukuho**	94.32±4.91a	10.18±1.01b	18.55±1.21a	17.37±2.04a	4.55±0.52a	64.27±7.04a	18.91±5.32b	34.96±2.98a	no
	**II-3-1a**	88.92±6.10b	11.15±0.73a	18.53±1.16a	15.81±1.20b	4.08±0.51a	60.67±5.07ab	24.58±4.94a	32.72±5.19a	yes
	**II-3-1c**	83.78±3.38c	9.36±0.63c	17.78±0.83a	18.02±1.66a	4.44±0.53a	58.00±8.11b	25.00±6.44a	23.83±2.10b	yes
	**BC1F2 +**	88.65±5.41b	11.14±0.74a	18.40±2.22a	15.58±1.24b	4.20±0.42a	60.20±4.80ab	24.10±3.41a	33.16±4.40a	yes
	**BC1F2 -**	94.45±4.50a	10.34±0.58b	18.73±1.19a	17.20±1.57a	4.45±0.52a	64.09±5.17a	18.27±3.44b	34.64±4.07a	no
**2016**	**Fukuho**	81.26±2.69a	9.57±0.59b	18.55±1.05a	18.41±1.54a	4.95±0.39a	69.80±4.53ab	13.75±3.46b	36.77±3.60a	no
	**II-3-1a**	74.36±4.28b	10.75±0.71a	18.31±1.32a	16.19±1.74b	5.15±0.38a	71.92±5.83a	17.15±3.18a	34.18±2.39a	yes
	**II-3-1c**	69.74±2.21c	8.49±0.40c	17.07±0.83b	18.97±1.30a	5.07±0.28a	65.14±4.07b	17.21±2.33a	26.38±1.15b	yes
	**BC2F2 +**	75.85±2.82b	10.75±0.49a	18.82±1.08a	16.58±1.03b	4.91±0.30a	71.91±10.27a	18.00±2.28a	34.95±3.32a	yes
	**BC2F2 -**	81.33±2.79a	9.66±0.36b	18.73±0.70a	18.37±0.80a	4.93±0.26a	69.73±5.09ab	13.73±2.15b	36.63±5.17a	no

Note: PH, Plant height; SL, Spike length; SNPS, Spikelet number per spike; SD, spikelets density; KNMS, Kernel number in the middle spikelet; GNPS, Grain number per spike; FTN, Fertile tiller number per plant; TGW, Thousand-grain weight; WGB, wheat glume bristles. ‘+’ indicated the progenies contained *A*. *cristatum* 1P chromatin; ‘-’ indicated the progenies contained no *A*. *cristatum* 1P chromatin. Significant differences in the mean are indicated at the P < 0.05 (lower-case letters) levels, based on Duncan’s multiple range tests.

## Discussion

### Potential value of production of wheat-*A*. *cristatum* disomic 1P addition line and 1P (1A) substitution line

Hybridization between wheat and its wild related species facilitates the utilization of exogenous desirable genes for wheat improvement. The disomic addition and substitution lines play bridge tools for the transfer of exogenous genes. To date, a large number of wild relatives of wheat have been used to establish disomic addition lines by hybridizing with wheat, such as *Thinopyrum intermedium* [31–33], *Secale cereale* L. [34], *Dasypyrum villosum* [35], and *Hordeum vulgare* [36–37]. Disomic addition lines could be used to locate genes on the alien chromosome and induce translocation line by *Ph1b* gene, gametocidal chromosome or ionizing irradiation. However, alien chromosomes might be lost in the disomic addition line during self-fertilization because of its limited stability. Since alien chromosomes in the substitution lines showed the homoelogous compensation with the substituted wheat chromosomes, so wheat breeders tend to use substitution lines in breeding program. To produce disomic substitution lines, disomic addition lines were always backcrossed by the responding nullisomic of common wheat. For example, wheat-barely 2H addition line was backcrossed by 2D nullisomic of wheat to get 2H-2D substitution line [38]. The monosome substitution line produced by the cross of disomic substitution line with common wheat could generate the translocation line due to misdivision. So the translocation lines were more stable and more useful for investigating homoeologous compensation between the alien segments and the deleted wheat segments, and for breeding new variety. In this study, a wheat-*A*. *cristatum* 1P addition line and a 1P (1A) substitution line was obtained respectively, which not only laid a good foundation for the research of genes on 1P chromosome but also provided materials for the production of translocation lines.

### Identification of *A*. *cristatum* 1P chromosomes in addition and substitution lines with molecular markers

Improvement of sequencing technologies and reduction of its cost laid good foundation for developing diverse functional molecular markers [20]. Molecular markers have potential to trace alien genes from wild species, identify homoeologous relationship between alien and wheat chromosomes and provide additional markers for comparative mapping. The transferability of wheat SSR markers was particularly important for genetic analysis, especially for the wheat-related species that had no genomic libraries [39]. Because EST sequences are always present in the expressed regions and conserved in gene transcripts, EST markers have been used to research homoelogous relationships among the species of *Poaceae*. For examples, wheat EST-SSR markers were used to determine the chromosome 5Ns in wheat-*Psathyrostachys huashanica* disomic addition line [40]. Four SSR markers showing length-polymorphisms between chromosomes 6B and 6G were obtained to monitor the 6B and 6G chromosomes in segregating generations involving the 6G (6B) substitution line [41]. In our study, three wheat SSR markers (cfe267, cfe77, and barc287) that could distinguish wheat chromosome 1A and *A*. *cristatum* chromosome 1P were screened. These three polymorphic markers could not only be used to monitor the 1P chromosome in segregating populations but also demonstrate the homoeologous relationship between 1A and 1P.

*A*. *cristatum* P-genome-specific markers have also made great progress due to the improvement of sequencing technologies. Wu et al. [23] obtained three SCAR markers using *A*. *cristatum* repetitive sequence. Han et al. [24] separated two *A*. *cristatum* repetitive sequence which could recognize *A*. *cristatum* chromosomes using DOP-PCR, and P-genome-specific STS primers were developed from *A*. *cristatum* transcriptome sequences [25]. *A*. *cristatum* STS markers were used to determine wheat-*A*. *cristatum* 6P addition line and locate genes on different chromosome segments [8, 15–17, 42]. In this study, 56 EST-STS markers specific to chromosome 1P were screened from *A*. *cristatum* transcriptome sequences that aligned on wheat homoeologous group 1. Those results demonstrated that the alien chromosomes in II-3-1a and II-3-1c were homoelogous group 1.

### Genes on chromosome 1P are potentially valuable in wheat breeding

Wild relatives of wheat are ideal gene pools for disease resistance, yield and quality. Many excellent alien genes have been transferred into wheat and played important roles in wheat improvement. In wheat production, the T1B.1R translocation line showing resistance against rust and powdery mildew, as well as good yielding capacity was efficiently used in wheat breeding programme [43–45]. *A*. *cristatum*, one important wild relatives of wheat, has various excellent genes. In this study, we found that multiple genes which controlling spike length, strengthen tillering ability, lower plant height and reduce spikelet density were located on chromosome 1P. The dwarfing genes Rht-B1, Rht-D1 and Rht8 had played great roles in deducing wheat plant height [46, 47]. Our results showed that the plants with chromosome 1P were significantly lower than those without chromosome 1P. It was predicted that chromosome 1P contained a novel dwarf genes which will provide new source for the dwarf gene family. Chromosome 1P also carried a gene (s) controlling the presence of bristles on the glume ridges which could be a typical characteristic to trace and identify 1P chromosomes. The wheat-*A*. *cristatum* 1P addition line and 1P (1A) substitution line will provide more useful genes and many great genetic diversity for wheat improvement.

## Supporting information

S1 Table Primer sequences of 56 1P-specific STS markers and 36 2P-specific STS markers. (DOC)

S2 Table Primer sequences of wheat chromosome 1A, 1B, and 1D-specific SSR and EST markers. (DOC)

## Abbreviations

CTAB: Cetyl-trimethylammonium bromide
DOP-PCR: Degenerate oligonucleotide primed-polymerase chain reaction
EST: Expressed sequence tag
FISH: Fluorescence in situ hybridization
Fukuho: Fukuhokomugi
GISH: Genomic in situ hybridization
RAPD: Random amplified polymorphic DNA
RFLP: Restriction Fragment Length Polymorphism
SCAR: Sequence characterized amplified regions
SLAF: Specific-locus amplified fragment sequencing
SSR: Simple Sequence Repeats
STS: Sequence tagged site
