# Supplementary material for: Chromosomal localization of genes conferring desirable agronomic traits from Agropyron cristatum chromosome 1P
Source: PLoS One. 2017 Apr 10;12(4):e0175265. doi: 10.1371/journal.pone.0175265 (PMC5386269; doi:10.1371/journal.pone.0175265)
Supplement: S1 Table — (DOC) [file pone.0175265.s001.doc]

**Table S1 Primer sequences of 56 1P-specific STS markers and 36 2P-specific STS markers**

| **Markers name** | **Location on wheat chromosome** | **Primer-F** | **Primer-R** | **Tm** |
| --- | --- | --- | --- | --- |
| Agc1048 | 1DL_2208503 | GGTACCTCAGCACCAAGGAA | TCGAGGAATCTGGGATGAAC | 60℃ |
| Agc1233 | 1AS_3297571 | ACGACACATCTCTCGATCCC | AAGTGCTGTGCGCTCCTACT | 60℃ |
| Agc13180 | 1AL_3844371 | GGGAAGGGTCAGACCAATTT | GGAAAGGCTGCGTACAAAAA | 60℃ |
| Agc13567 | 1BL_3878546 | AGACACAACTCACCTTGGGG | GACTCTAGGCTCGCCATCAC | 60℃ |
| Agc14448 | 1AS_3293400 | TTGCAGATGTTGAGGAGCTG | TAGTTTGTGCCCCAGGAGTC | 60℃ |
| Agc17266 | 1BS_3400391 | GCCATGATTCTCCAGTTGGT | CAAACCTGCTTCCAACCATT | 60℃ |
| Agc17406 | 1DL_2287331 | TAGCCGGGGAAAGAAAAGAT | GGCATGGACCTGAGCTAGAG | 60℃ |
| Agc20757 | 1DS_1879687 | GTGTTTTTGGTGCTCGTCCT | AGATGGAAGAGAACGGGAGG | 60℃ |
| Agc21000 | 1AL_3840877 | GACCCTGGAATGCTGTCAAT | GCGTGCTCTCTATGTAGCCC | 60℃ |
| Agc21222 | 1DL_2248694 | CCACTCTAGCCGGTCATTCT | ACCTAGCCAAGTCGCTTTCC | 60℃ |
| Agc21228 | 1AS_2470305 | TCTCCCTTGGAAAAGAAGCA | TGGGGCCGACTAATAAAGTG | 60℃ |
| Agc2142 | 1BL_3892254 | CGGATACGCAGCGAAATTAT | TCTCCAGTTCAACCCAAAGG | 60℃ |
| Agc22246 | 1AL_3900709 | CTCTCGGAGGTTGGCTTGT | TCTCCATCCTCGTGTTCAGC | 60℃ |
| Agc23234 | 1BS_3472667 | AAACACGTGCCCGAAGTAAC | CATATGATCCAGGGTTTGGC | 60℃ |
| Agc23256 | 1BS_3422051 | CCCACACAGTTCCCTCCATA | TTGTGATAGCGAGGTGCTTG | 60℃ |
| Agc24325 | 1DS_1881199 | ACCATCAACACCCACAAGGT | TGATGTTGCTTGTAGCACCC | 60℃ |
| Agc2638 | 1DS_1887051 | GACGAGAAAGCCATGGTGAT | GGGTGGAGAGAATTGCAAGA | 60℃ |
| Agc26797 | 1BS_3447018 | CGCCAATAATGGAGGTTTGT | TCGATACTACACACGGACGC | 60℃ |
| Agc27311 | 1AL_3876661 | CGAATTGCCCTTGATAGGAA | ATGTCAAGCGCATCTTTGTG | 60℃ |
| Agc27774 | 1BS_3458802 | CTGTGGAGAGGTGGGTGAGT | CTCCACTGGACTCGACAACA | 60℃ |
| Agc28830 | 1BL_3820055 | TCGACCATATCCTCACCCAT | GTTGGGTTGGAGCGTTAGAA | 60℃ |
| Agc29588 | 1BS_3475762 | GGAGATCACTTAGCGGTGTCA | GTAGCAAGCTCTTGTTGCCC | 60℃ |
| Agc34192 | 1AS_3281105 | GTTCTTCCACCTCCCCGT | GTACCCTTCGTAGGTCGCC | 60℃ |
| Agc34393 | 1AL_3897631 | TTGTAAATTTCGCTCCCTGC | GTTTGCATGTGCTAAGCGAA | 60℃ |
| Agc35212 | 1BS_3477327 | CCTAAGCTTCTTCGTGTCCG | TAGCGAATGACGACCAACAG | 60℃ |
| Agc38044 | 1AS_152605 | CATTGCTCCCGTACTTCCAT | TGGATGTATGGTGATGGTGG | 60℃ |
| Agc3994 | 1AS_3283712 | GACCAGAAAAGCACTCCAGC | CCAGGAAGACCTTACGTCCA | 60℃ |
| Agc42929 | 1BL_3803462 | GAAGAGCGACAGAACACCAA | TTTATGCTTGTGCAGGTGGA | 60℃ |
| Agc4379 | 1AL_3916263 | GACAGAGGGGATAACGGGAG | GCTCCACCCACCACTAACTC | 60℃ |
| Agc46715 | 1AL_3890824 | GTATCGTCTCCCTCAGCCAC | TGCACATCATTATCAGGGGA | 60℃ |
| Agc4677 | 1AL_3918481 | CGCATCATCCTCGTCTTGT | GACAACTTCCGCTTCTGCTC | 60℃ |
| Agc508 | 1BS_3478977 | GTCTTTCCCAGATGGTCCAA | TTGTCGGCAATGTGCATTAT | 60℃ |
| Agc50996 | 1AS_3285956 | GGTGGTGAGGAAGATGTTGG | ACCACTTCCGCATTCAAAAC | 60℃ |
| Agc52299 | 1DL_2239738 | TGATCGCAAGCTACTACGGA | TAAAGTAAGCATGCGACCCC | 60℃ |
| Agc55475 | 1BS_3472667 | CATGCACGTGAGCACTAGGT | GTAGGCCCTTTGTCTGTGGA | 60℃ |
| Agc6043 | 1AL_3880852 | CTCCAGCTTCAGTTTCAGCC | AGGCCAAGAAAAGAGAAGCC | 60℃ |
| Agc6184 | 1DL_239589 | GATCCGCTACTGTTTCTCGC | TCTCTCGCCTACTGCCTCTC | 60℃ |
| Agc627 | 1DL_1459577 | TCTGTTCTTCGTCCGTGTTG | GAGACCGGGGACAGTAATGA | 60℃ |
| Agc64023 | 1BS_3438578 | TGAAGTATCCAGAGCCTGCC | CACGACGTCTTCACACTGCT | 60℃ |
| Agc65293 | 1BL_3870897 | TGAGTGACAGACATGGGTGG | GTGCATCCATCACTCCAGC | 60℃ |
| Agc65432 | 1DL_2262979 | TCTGTGTGGCATCAAATCGT | GTTTGCTGAAAGTGCTGTGC | 60℃ |
| Agc67608 | 1AL_3975934 | CTGCGTGGATCTTGATGCTA | GTTCAGAAGGGGACGATTCA | 60℃ |
| Agc68263 | 1DL_1755123 | CATCTTGTGTGTCGCTCCAT | GATAGCCCCTTTCACGTTCA | 60℃ |
| Agc68983 | 1DL_2285087 | ACACCCTGAATCCTCAATGC | CAAGCCAGTCAAGAACACGA | 60℃ |
| Agc69388 | 1BS_3451628 | TTTAGACGGCGAGCATTCTT | ACAAGTTCATTCGCCACCTC | 60℃ |
| Agc7191 | 1BL_3891989 | CGTGAAGATCACGAAGCAAA | CAGGACACCAAGTGCAACAC | 60℃ |
| Agc71982 | 1DL_2284594 | GACCCAAGACGTGAGGTTGT | CTTCGAGAGCATCCATGACA | 60℃ |
| Agc8189 | 1AS_3268585 | CGAGGAAGATCCTGGTGGTA | ATCATCGGCGTTCTGGTTAC | 60℃ |
| Agc904 | 1BL_3897372 | TCTCACGAGTGTTGGTCGAG | TACTGACGCCACCTCACAAG | 60℃ |
| Agc9315 | 1BL_3882508 | CCTGGTCTTTTTGTTGGCAT | TACTGGGAACTTCTCGGGTG | 60℃ |
| Agc9387 | 1BL_3858385 | GCAGCAGCTAGTGGTAGCGT | CCGGTGCTGGTAGCTAGTGT | 60℃ |
| Agc9509 | 1DL_1127622 | TTGGATAAGGAGCAAATGGC | TGACACGGAGATGGTGCTAA | 60℃ |
| Agc9626 | 1AL_3978429 | ATAAGACTTGTGGGCATCGG | CACGGTTCAGCTTGTTGATG | 60℃ |
| Agc9770 | 1AL_3910842 | CGTGGAACATTACAAATGCG | TAGCTTAGCGCACCGAGAAT | 60℃ |
| Agc9865 | 1BL_3868625 | CCAATCATACGCACATCGTC | TCAGTTTTGGTGATCGGTGA | 60℃ |
| Agc9953 | 1BL_3893398 | AGGAGCCTTCCCTAAAGTCG | GCTTCTAGTGGGCTGAATGC | 60℃ |
| Agc10086 | 2DL_9873625 | CGTCTTAGGAGCGTGGACTC | ATCAGCCTAATTGGTGCGTT | 60℃ |
| Agc10386 | 2DL_6242415 | AAGTGCTGATCCAACCAACC | GTTCATGACCGTCTCGTCCT | 60℃ |
| Agc10705 | 2BS_5210335 | ACATGGGGAGCTAGTTGTGC | TACTCTGAAGCGCCCATACC | 60℃ |
| Agc10757 | 2DS_5293062 | CACTGTGTCGGCAGAAGGTA | AGGCACTAGGATTGTGGGTG | 60℃ |
| Agc10935 | 2BL_7954970 | AACATCAACAACCCGGAGTC | GACGGTCGAGTCATACGGTT | 60℃ |
| Agc10944 | 2AL_6418689 | AACATTGTTGGGTCTTGGATG | ATTGGGCATGTTTCCAGTTC | 60℃ |
| Agc11973 | 2AL_6395225 | AGTGAGGTGGTGCTGGATTC | GCGGCAATGAGTGACTGTAA | 60℃ |
| Agc12244 | 2AL_6329982 | GTATGGCCTTGCAGGTGACT | ATGACAAACAGGGGTGGTGT | 60℃ |
| Agc19918 | 2AL_6315374 | GCGAGAGCGGGTACTACAAG | CACTTGTTTTTGACATGCGG | 60℃ |
| Agc24824 | 2DL_9807167 | AGCTACACATGCACCAGCAC | GCCTCCACCACTGCTTAGTC | 60℃ |
| Agc25953 | 2AS_5270580 | AGAAGCACTTGCCTTTGGAA | GAACTGATTGACCTCCCAGC | 60℃ |
| Agc26300 | 2DL_9823151 | GGTCGGTAGGGGGTCTGTAT | GCGCAGGGTATATTGTGGTT | 60℃ |
| Agc26312 | 2DL_9829993 | GGATGAGGAGGAGAGGAAGG | CTTCTCACAAGAAGACGCCC | 60℃ |
| Agc28421 | 2DL_9887026 | CAACATGCCCACATGATCTC | GGCAGATTGTTTTGGTTTTGA | 60℃ |
| Agc29684 | 2DL_9860709 | GTGTGAACACGTTGGACTGG | CTCACTAGCGGTAGGGGATG | 60℃ |
| Agc29835 | 2AS_5189528 | TGCACCAATCATCGGTCTAA | CAACACCACTCTTCGTTCCA | 60℃ |
| Agc31105 | 2BS_5176869 | GGCATGGGAGTAAGGAGACA | AGTCGTATGCTCTTGGCGAT | 60℃ |
| Agc31114 | 2DL_9832876 | GTTGAAGAAGACGCAAGGCT | GCATGGCAATGAGAATAGCA | 60℃ |
| Agc31136 | 2BL_8022858 | ACCTAGCGCTTTCCATTTGA | GAAAACAGGCAGGAGCAGAC | 60℃ |
| Agc31586 | 2AL_6372939 | GCGGAGCTTGAAAGAAAATG | TCTGTGCTCTCCATTTGTGC | 60℃ |
| Agc32533 | 2AL_6384177 | AAGCCAAAGATGGTAGCCCT | GGGAAGTCCAGTTCATCCAA | 60℃ |
| Agc33175 | 2DL_9835212 | TACTCTGGTAGCTTCCCCCA | GGTTCTGCCCACAGTTGATT | 60℃ |
| Agc3398 | 2BL_8042453 | CAGAACGGGAGGAGGTATCA | GAAGCTACCTGAGGCTGCAC | 60℃ |
| Agc3425 | 2DS_5343224 | GGCCTCTCTGACGATGTGAT | TCTGCTTGGGTACTGATCCC | 60℃ |
| Agc35 | 2BS_5213997 | CTGGAAAAAGAGCTGCAAGG | TTGCCGTGACTGAATAATGC | 60℃ |
| Agc353 | 2DL_9909176 | TTTGTTGTATCCGGGCAAAT | CAAGCAAAAACGCTCATCAA | 60℃ |
| Agc3650 | 2BS_5227346 | GATCAGTGGTTGGGAGGAAA | TTGCAGAGGACCTTTGTGTG | 60℃ |
| Agc368 | 2AL_6400376 | GCCACATGAAGACATCGCTA | TCTCGCTAATCTGGTGTTGC | 60℃ |
| Agc4115 | 2DL_9872141 | TATCCTCAGTTCCCCGACAG | TTGTGCTGTGCGTGTGTAAA | 60℃ |
| Agc4244 | 2BS_5202139 | CTACTGCGAGTTTGGCGATT | AGGAACCAGACACACGATCC | 60℃ |
| Agc4250 | 2AL_6354503 | TTGTGGGCTCAATCATTTCA | CTGGCAAGAAGCATGGATTT | 60℃ |
| Agc50622 | 2BL_7998993 | GTTCTCAAGGTCTGGTCCGT | GAGCATATTCATCATGGGCA | 60℃ |
| Agc52100 | 2AL_6411040 | CGACTGGGAAGTATCGGGTA | AGTCACCGAGCAACTCGTTT | 60℃ |
| Agc54078 | 2DL_9909807 | GGCGACTTAGTGCATTGGAT | AATGGAGTTGCCTACATCCG | 60℃ |
| Agc5658 | 2BL_7965965 | GCCTCTTATTTGTCGGTGGA | GGATGAAAACTGCTTGAGGC | 60℃ |
| Agc924 | 2BS_5167717 | CAAGAGGCAAAACACAAGCA | GGCCAGAACAGAATGCTCTC | 60℃ |
