# Supplementary material for: Chromosomal localization of genes conferring desirable agronomic traits from Agropyron cristatum chromosome 1P
Source: PLoS One. 2017 Apr 10;12(4):e0175265. doi: 10.1371/journal.pone.0175265 (PMC5386269; doi:10.1371/journal.pone.0175265)
Supplement: S2 Table — (DOC) [file pone.0175265.s002.doc]

**Table S2 Primer sequences of wheat chromosome 1A, 1B,and 1D-specific SSR and EST markers**

| **Marker name** | **Chromosome**  **location** | **Primer-F** | **Primer-R** | **Tm** |
| --- | --- | --- | --- | --- |
| Barc287 | 1A | CGGATGGGTTACTTACTTAGGATG | CGCAACTCCATTTCAGAATCATT | 55℃ |
| Cfe267 | 1A | GACGTAGACCTTGACCGCC | CATTCCATCTCGAACCCATT | 55℃ |
| Wmc278 | 1A | AAACGATAGTAAAATTACCTCGGAT | TCAAAAAATAGCAACTTGAAGACAT | 55℃ |
| Barc9 | 1AS | GCGGTCGGTGTCTCCAGTTTTTTTATCA | GCGACATGCGGACAGTATTTAAATTTC | 55℃ |
| Barc120 | 1A | CCCCCTCTCTTCCTCAT | ATATAGCTCCCCCATTTCCT | 55℃ |
| Barc263 | 1AS | GGAAGCGCGTCAGCACTAGGCAAC | GGCTTCTAGGTGCTGCGGCTTTTGTC | 55℃ |
| Cfa2135 | 1A | TGCCTAAATCTAAATGCCCG | GGATAATGTGCATGTTCACCG | 55℃ |
| Cfe189 | 1A | ATACAGAACCGGACACGAGG | CACCAAGGACAACACCACC | 55℃ |
| Cfa2219 | 1A | TCTGCCGAGTCACTTCATTG | GACAAGGCCAGTCCAAAAGA | 55℃ |
| Barc17 | 1AL | GCGCAACATATTCAGCTCAACA | TCCACATCTCGTCCCTCATAGTTTG | 55℃ |
| Barc99 | 1DL | CGCATTCTTTCGCATTCTCTGTCATA | CGCATACTGTGTCGTGTTCCTGGTTTAGA | 55℃ |
| Wmc183 | 1A | CAGAAACGGCTCAACTTAACAA | TCTGATCTCGTGATCAGAATAG | 55℃ |
| Wmc716 | 1A | CATTTATGTGCACGCCGAAG | CCATAAGCATCGTCACCCTG | 55℃ |
| Wmc24 | 1A | GTGAGCAATTTTGATTATACTG | TACCCTGATGCTGTAATATGTG | 55℃ |
| Wmc120 | 1A | GGAGATGAGAAGGGGGTCAGGA | CCAGGAGACCAGGTTGCAGAAG | 55℃ |
| Cfe77 | 1A | AGCTCTCGGCTCACCTCTC | CAGTGTCACCGGCTCGTC | 55℃ |
| Cfe103 | 1A | TCGCACCAGCTACAACACTC | CCATGATTTGTCCGCTTCTT | 55℃ |
| Wmc237 | 1A | TAAGTGAATCGATGGGCGTGGA | CAGTGTGCAGGGGAAGCAGTGT | 55℃ |
| Xgwm164 | 1A | ACATTTCTCCCCCATCGTC | TTGTAAACAAATCGCATGCG | 55℃ |
| Cfd170 | 1B | CTGTCGGACGACGACGA | CATCCTCTTGACGCCGCCGC | 55℃ |
| Xgwm458 | 1D | AATGGCAATTGGAAGACATAGC | TTCGCAATGTTGATTTGGC | 55℃ |
| Xgwm124 | 1B | ACTGTTCGGTGCAATTTGAG | GCCATGGCTATCACCCAG | 55℃ |
| Xgwm153 | 1B | GATCTCGTCACCCGGAATTC | TGGTAGAGAAGGACGGAGAG | 55℃ |
| Xgwm258 | 1B | AGGGAAAAGACATCTTTTTTTTC | CGACCGACTTCGGGTTC | 55℃ |
| Xgwm268 | 1B | AGGGGATATGTTGTCACTCCA | TTATGTGATTGCGTACGTACCC | 55℃ |
| Xgwm413 | 1B | GATCGTCTCGTCCTTGGCA | TGCTTGTCTAGATTGCTTGGG | 55℃ |
| Cfd27 | 1D | GCAGCAAGATCAAATCGACA | ACTGAGGACTTGGTGCCATC | 55℃ |
| Cfd32 | 1D | CAACACAACCACAATTTCCG | CTCAGGGAGGTCATGCAGAG | 55℃ |
| Cfd61 | 1D | ATTCAAATGCAACGCAAACA | GTTAGCCAAGGACCCCTTTC | 55℃ |
| Cfd63 | 1D | GAGGATGTTGAGGACC | GAGAGAGGCGAAACATGGAC | 55℃ |
| Cfd83 | 1D | AAGGATGGAGAGGACCCCTA | GGAGGTGGAGCAACCTATCA | 55℃ |
| Cfd92 | 1D | CTTGTTGATCTCCTTCCCCA | TTCTCTCATGACGGCAACAC | 55℃ |
| Cfd282 | 1D | TCTCATCCCTGTTCCTCTGC | GTCGACGTCTGCACATTGTT | 55℃ |
